# Effects of Hydrogen-Rich Saline on Taurocholate-Induced Acute Pancreatitis in Rat

**DOI:** 10.1155/2013/731932

**Published:** 2013-07-28

**Authors:** De-qing Zhang, Huang Feng, Wei-chang Chen

**Affiliations:** Department of Gastroenterology, The First Affiliated Hospital of Soochow University, Suzhou, Jiangsu 215006, China

## Abstract

Oxidative stress plays an important role in the pathogenesis of acute pancreatitis (AP). As an ideal exterminator of poisonous free radicals, hydrogen can clearly reduce the degree of oxidative damage caused by severe acute pancreatitis (SAP) and lessen the presence of inflammatory cytokines. The aim of this study was to investigate the effects and mechanism of hydrogen-rich saline on SAP in rats. Serum TNF-**α**, IL-6, and IL-18 and histopathological score in the pancreas were reduced after hydrogen-rich saline treatment. Malondialdehyde (MDA) and myeloperoxidase (MPO) contents were obviously reduced, while superoxide dismutase (SOD) and glutathione (GSH) contents were increased after hydrogen-rich saline treatment. The expression of mRNA of tumor necrosis factor-**α** (TNF-**α**) and intercellular adhesion molecule-1 (ICAM-1) in the pancreas was reduced in hydrogen-rich saline treated group. In conclusion, intravenous hydrogen-rich saline injections could attenuate the severity of AP, probably via inhibiting the oxidative stress and reducing the presence of inflammatory mediators.

## 1. Introduction

Acute pancreatitis (AP) is a common disease in the department of gastroenterology, but the pathogenesis of acute pancreatitis is still not fully explained. In recent years, attention was drawn to the role of oxygen radicals and inflammatory mediators in acute pancreatitis [1]. Studies show that systematic inflammatory response syndrome (SIRS) may occur after the initial inflammatory responses during the onset of pancreatitis [2, 3]. During the inflammatory response process caused by AP, insufficient blood volume, hypoxia, and discharge of large amounts of inflammatory mediators brought on by microcirculation in the pancreatic tissue will activate neutrophils and cause production of large quantities of oxygen radicals, which may heighten the degree of inflammatory response, so far as to damage internal organ function [4]. The severity of AP might be determined by the events that occur subsequently to acinar cell injury, including the activation of tumor necrosis factor such as TNF-*α*, the recruitment and activation of inflammatory cells, and the generation and release of cytokines and other inflammation mediators [5–7]. During AP, oxygen free radicals play an important role in triggering subsequential lesions in the pancreas, which were produced in damaged acinar cells as well as activated neutrophils and macrophages [8, 9]. Hydrogen molecules act as a new kind of selective antioxidant, which can significantly reduce oxidative damage to the brain, liver, heart, small intestine, and so forth, caused by ischemia reperfusion, and can effectively suppress the inflammatory damage caused by colonitis and drug-induced hepatitis [10–15]. However, the effect of hydrogen-rich saline in SAP is still not fully clarified. This animal experiment was designed to study the protective effects of hydrogen-rich saline on AP in rats, for introducing a new line of thought into the treatment of SAP. 

## 2. Materials and Methods

### 2.1. Preparation of Hydrogen-Rich Saline

High pressure hydrogen gas (0.4 MPa) was mixed with 0.9% sodium chloride for six hours to attain appropriate concentration (>0.6 mmol/L). The solution was gamma-ray sterilized and preserved at 4°C gas in an aluminum bag with no dead volume chromatography testing measured the hydrogen density at 0.75 mmol/L, approaching 0.6 mmol/L, an effective hydrogen-treatment concentration [16]. Moreover, hydrogen-rich saline was freshly prepared before the treatment group received an injection of hydrogen-rich saline in our experiment.

### 2.2. Animals

Forty-five specific pathogen free Sprague-Dawley (SD) rats (8–10 w old males, *n* = 15/group) were selected from the Experimental Animal Center of the Soochow University. The animals were kept in a conventional room with a 12 h light-dark cycle at constant temperature. All procedures were performed in accordance with the guidelines of the Institutional Animal Care and Use Committee of the Soochow University. Rats were randomly divided into 3 groups (15 in each group): control group, model group and treatment group.

### 2.3. Induction of Pancreatitis

All rats were fasted 12 hours prior to surgery, with free access to water. Rats were anesthetized by intraperitoneal injection of 2% sodium pentobarbital (40 mg/kg); then the abdominal area was sterilized. Then the common bile duct was accessed through a midline incision and entered at an antegrade direction using PE-10 tubing (Fisher Scientific, Pittsburgh, PA, USA). This ensured that the proximal end of the tube was beyond the ampulla of Vater in the duodenum. The bile duct was then ligated to forestall the flow of bile, and 4% sodium taurocholate (1 mL/kg) in sterile saline was continuously infused into the pancreatic duct at a rate of 0.1 mL/min over 10 min. In the control group, incisions were closed immediately following the turning over of the pancreas; no other treatment was given. One hour after the induction of AP, rats in the treatment group received an injection of hydrogen-rich saline 5 mL/kg while the model group was treated with normal saline solution 5 mL/kg all by tail vein. Animals were sacrificed under inhalation anesthesia 24 h after the induction of pancreatitis, and the blood or pancreatic tissue samples were collected for analysis.

### 2.4. Histopathological Analysis

Paraffin-embedded tissue samples were sliced and stained by hematoxylin and eosin (HE). Histopathological changes of the pancreas were evaluated according to a scoring system previously described [17]. The severity of acute pancreatitis was blindly graded by a semiquantitative assessment of edema, inflammatory cell infiltrate, and acinar necrosis, based on the following criteria: edema: 0 = absent, 1 = focally increased between lobules, 2 = diffusely increased between lobules, 3 = acini disrupted and separated. Inflammatory cell infiltrate: 0 = absent, 1 = rare or around ductal margins, 2 = in the parenchyma (<50% of the lobules), and 3 = in the parenchyma (>50% of the lobules). Necrosis: 0 = absent, 1 = architectural changes, 2 = pycnotic nuclei, and 3 = focal necrosis (<10% of the parenchyma); 4 = diffuse parenchymal necrosis (>10% of the parenchyma). Histological score: sum of edema, inflammatory cell infiltrate and acinar necrosis scores.

### 2.5. Assay of Inflammatory Mediators and Cytokines

Blood samples were collected in ethylenediaminetetraacetic acid-Na tubes and centrifuged at 2000 g for 10 minutes at 4°C immediately after collection; the plasma was separated by using sterile pipettes and stored at −80°C until assayed. Concentrations of TNF-*α*, IL-6, and IL-18 in plasma were measured in picograms per milliliter by commercially available enzyme-linked immunosorbent assay kits (Shanghai XiTang Biotechnology Corp., Shanghai, China). 

### 2.6. Measurement of MDA, MPO, and GSH Levels in Pancreas

The pancreatic tissue was homogenized immediately on ice in 5 vol of normal saline. The MDA, MPO, and GSH were detected using MDA, MPO, and GSH commercial assay kit (all from Nanjing Jiancheng Corp., Nanjing, China), following the manufacturer's recommendations.

### 2.7. Detection of SOD Activity in Pancreas

The activity of SOD in pancreas was measured using a commercial assay kit purchased from Nanjing Jiancheng Corp (Nanjing, China), following the manufacturer's instructions. This assay kit uses a tetrazolium salt for detection of superoxide anions generated by xanthine oxidase and hypoxanthine. These superoxide radicals oxidize hydroxylamine and lead to formation of nitrite, which reacts with naphthalene diamine and sulfanilic acid to produce a colored product. SOD in the sample reduces the overall superoxide anion concentration, thereby lowering the colorimetric signal and absorbance at 550 nm. One unit (U) of SOD was defined as the amount of enzyme needed to produce 50% dismutation of superoxide radical. The total tissue protein concentration was determined by a commercial kit (Nanjing Jiancheng Corp., Nanjing, China), and the activity of SOD was expressed as U/mg of protein.

### 2.8. Detection of TNF-*α*mRNA Expression in Pancreas

The expression of TNF-*α*mRNA in the pancreatic tissue was detected by using semiquantitative RT-PCR. Briefly, total RNAs in the pancreatic tissue were extracted using TRIZOL reagent (Gibco, Grand Island, USA) and tested for absorbance with a spectrophotometer set at 260 nm~280 nm, as well as RNA concentration and purity. Total RNA was converted to cDNA with random primers, at which point a TNF-*α* primer was used for amplification, with *β*-actin serving as internal control. The upstream sequence of TNF-*α* primer forward sequence was 5′-CCAACAAGGAGGAGGAGAAGT-3′, while the downstream sequence was 5′-GTATGAAGTGGCAAATCG-3′, and the amplified fragment was at 323 bp. The *β*-actin upstream sequence forward primer was 5′-CACGATGGAGGGGCCGGACTCATC-3′, and the downstream sequence was 5′-TAAAGACCTCTATGCCAACACAGT-3′, and the amplified primer fragment was 241 bp. During PCR the annealing temperature was 54°C and the cycling number was 38.

### 2.9. ICAM-1 Immunohistochemical Staining

ICAM-1 immunohistochemistry was performed on formalin-fixed paraffin embedded tissue. Sections of 5 *μ*m were deparaffinised with xylene and rehydrated in a series of graded ethanols to distilled water. Immunohistochemical staining of ICAM-1 was performed using the Bond Polymer Refine Detection of Bond Automated Immunohistochemistry system (Leica Microsystems, Inc., Bannockburn, IL). Briefly, formalin fixed, paraffin embedded 5 *μ*m thick sections were deparaffinized, rehydrated, and heated at 100°C using citrate buffer (pH 6.0) for 5 min. Sections were subjected to sequential incubations with endogenous peroxidase block, primary antibody (at 1 : 2000 dilution), postprimary (secondary antibody), polymer (tertiary antibody), diaminobenzidine (DAB), and hematoxylin for 5 min (Bond Polymer Refine Detection; Leica Microsystems), respectively. Two pathologist blinded to the diagnosis performed the immunohistochemical analysis as previously described [18]. The numbers of ICAM-1 positive cells (stained in brown-yellow and located in the cell membrane and the cytoplasm) were counted in five high-power fields in each section. The evaluation criteria were as follows: (1) staining intensity was evaluated and assigned a score of 0–3+ : 0 = negative, 1+ = weak, 2+ = moderate, 3+ = strong. (2) Immunoreactivity score was assigned based on the proportion of positive cells over total cells (percent positivity) ranging from 0% to 100% on a scale of 0–3 : 0 = 0% positive cells, 1 = 1–25% positive cells, 2 = 26–50% positive cells, and 3⇒60% positive cells. The immunohistochemical scoring was then assigned by multiplying the staining score and immunoreactivity score; the score thus ranged from 0 to 9.0.

### 2.10. Statistical Method

All experimental values are expressed as mean ± standard deviation (SD). Statistical analysis was made by analysis of variance (ANOVA) followed by LSD-t post hoc test for multiple comparisons using the computer statistical package SPSS 16.0 (SPSS Inc., Chicago, IL, USA). The Kruskal-Wallis test was used to evaluate the differences in categorical values followed by Mann-Whitney *U* tests as a post hoc test. *P* value of <0.05 was statistically significant.

## 3. Results

### 3.1. Hydrogen-Rich Saline Attenuates the Severity of Pancreatitis

**Figure 1:**
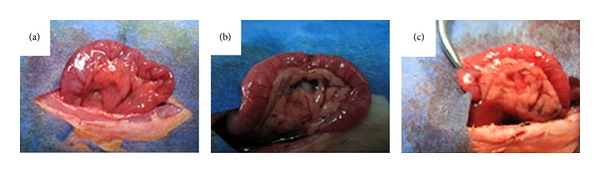
Gross pathological changes in pancreases. (a) Control group, (b) model group, (c) treatment group. The pancreases of the model group were obvious swelling and hardening, while in the treatment group, and they exhibited limited congestion edema. The pancreases of the control group were soft and smooth without any morphological change.

**Figure 2:**
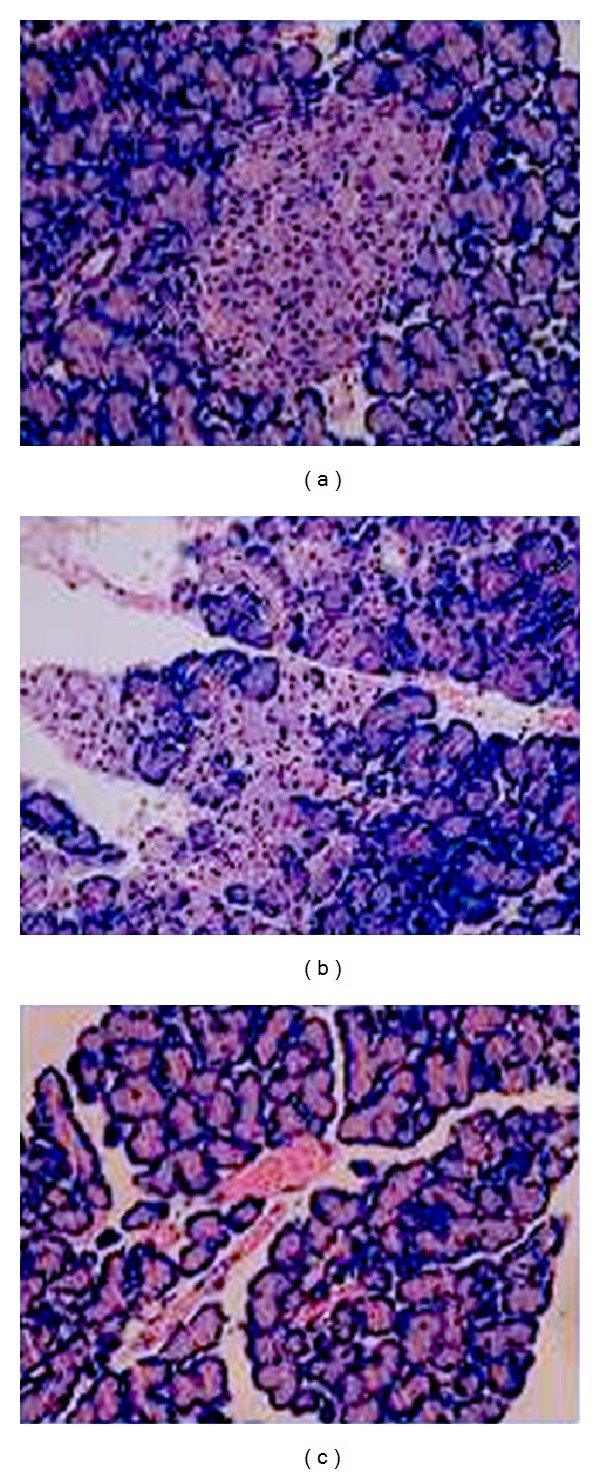
Pancreatic H&E stained sections. (a) control group; (b) model group; (c) treatment group. The model group exhibited extensive hemorrhaging, necrosis and lots of inflammatory cells, while control group the change was not remarkable. The control group exhibited few pathological changes, pancreatic tissue showed no inflammatory permeation.

The pancreas of the control group were soft and smooth without any morphological changes (Figure 1(a)). While in the model group, the pancreas exhibited obvious swelling, as well as hemorrhaging of ascitic fluid (Figure 1(b)). After dissection, focal necrosis was observed. However, limited congestion edema was exhibited in the treatment group, with less ascites in the abdominal cavity (Figure 1(c)). Compared with the control group, extensive hemorrhaging and necrosis were exhibited in the model group, with massive inflammatory cell infiltration and lobular damages. Of these the most important were the neutrophils and mononuclear cells; remaining cells were all significantly swollen. While the pathological changes in the treatment group were obviously ameliorated and the pathology scores in the treatment group were significantly lower than those of the model group (7.5 ± 0.9 versus 4.1 ± 0.3, *P* < 0.05, Table 1, Figure 2(c)).

### 3.2. Hydrogen-Rich Saline Reduces the Secretion of Proinflammatory Cytokines in the Pancreas

Levels of IL-6 and IL-18 in the model group were clearly higher than those of the control group (*P* < 0.05), and levels in the treatment group were clearly lower than levels in the model group (*P* < 0.05, Table 1). Levels of TNF-*α* and TNF-*α* mRNA expression of the model group were significantly higher than those in the control and treatment groups (*P* < 0.05, Table 1).

**Figure 3:**
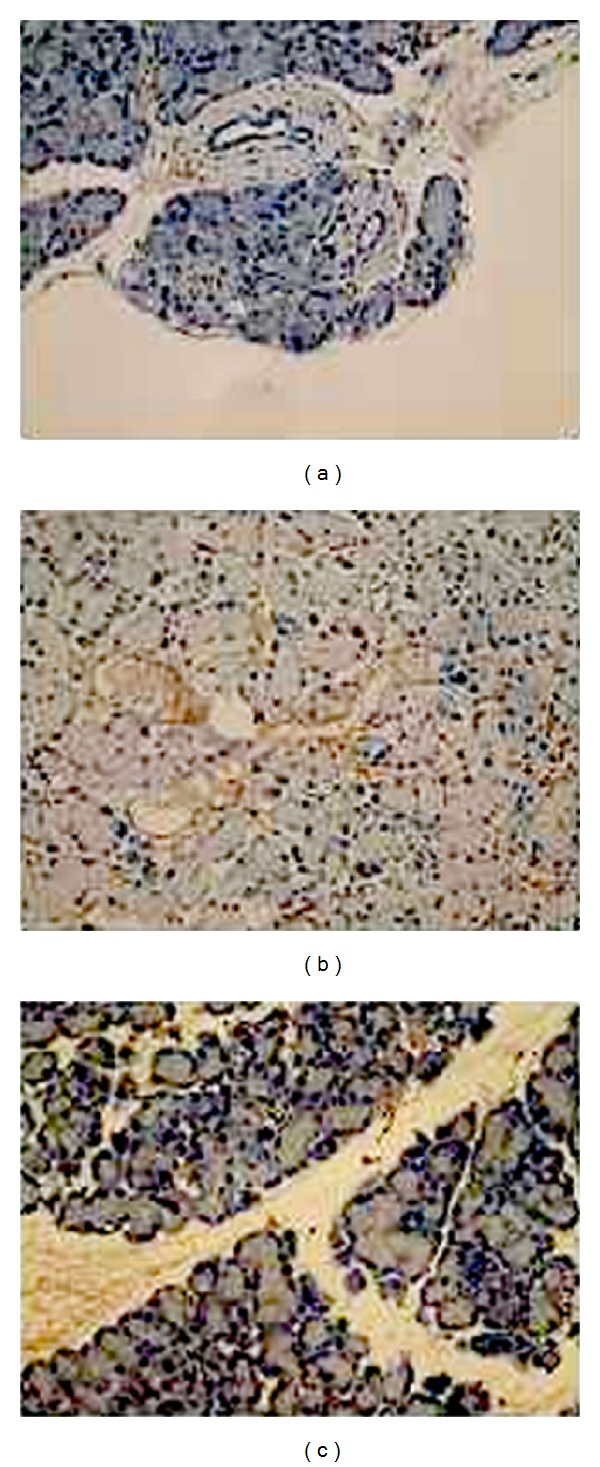
ICAM-1 immunohistochemistry in pancreases samples. (a) Control group, (b) model group, and (c) treatment group. In weakly staining, positively stained cells showed a brown-yellow pigment in control group. The model group exhibited the strong positive expression of ICAM-1 with lots of brown-yellow stained particles in cells, while in treatment group the change was not remarkable.

ICAM-1 level in pancreatic tissue of the model group was significantly higher than that of the control. ICAM-1 level in the pancreatic tissue of the treatment group were significantly lower than levels in the tissue of the model group (2.27 ± 0.41 versus 6.41 ± 0.68,  *P* < 0.05, Table 1, Figure 3).

### 3.3. Hydrogen-Rich Saline Reduces the Oxidative Stress in the Pancreas

MDA and MPO levels in the pancreases of model group were far higher than those of the control group, while SOD and GSH levels were significantly lower than those of the control group. MDA and MPO levels in the pancreatic tissue of the treatment group were significantly lower than levels in the tissue of the model group, while SOD and GSH levels were clearly higher than those of the model group (*P* < 0.05, Table 1).

## 4. Discussion

The pathogenesis of SAP is not completely understood. Researches show that the pathologic development of SAP is closely associated with the mediation of oxygen radicals and cytokines [19, 20]. During the pathogenesis of SAP, the level of oxygen radicals in the pancreatic tissue increases significantly and these oxygen radicals will attack the unsaturated fatty acids located in the cell membrane creating lipid peroxide [21]. 

In the early phase of SAP, large amounts of oxygen radicals are created and further damage capillary endothelial cells, which in turn increases microcirculatory disturbance. In the late-stage development, chemotaxin and activation of white blood cells are the primary pathophysiologic effects of SAP, which are concurrent with increased oxygen radical production and the deterioration of pancreatic acinar cells [22]. MDA is an end product of unsaturated lipid peroxide reaction, and MPO is an important peroxide enzynme produced by azurophilic granules in neutrophils, that is even used as a specific physical indicator of neutrophils infiltration, and can reflect the severity of inflammation [23, 24]. The levels of MDA measured in the pancreases of SAP rats in the model group was considerably higher than that in control group rats, while hydrogen-rich saline treatment significantly reduced the MDA level. After being injected with hydrogen-rich saline, MPO activation was noticeably reduced indicating the reduction of neutrophil permeation.

At present, inflammatory mediators play a pivotal role in the early stage of pancreatitis. Among these, TNF-*α* is thought to be a trigger of cascading inflammatory reactions in SAP and functioned on many kinds of cells, at the celluar and subcelluar levels [25]. Interleukin-6 (IL-6) is also an important mediator during inflammatory response, as a part of acute reaction and inducing ICAM-1 expression, and forms a network with TNF-*α* [26]. Induced by activated mononuclear macrophages during SAP, TNF-*α*, and IL-6 are thought to contribute to the onset of this disease. In our study, serum TNF-*α* and TNF-*α*mRNA expression in the pancreas was reduced after hydrogen-rich saline was administrated in the pancreatitis induced by taurocholate. During onset of SAP, IL-18 can stimulate production of IFN-*γ* [27]. Research by Ueda et al. [28] shows that the presence of IL-18 during SAP correlates to higher rates of multiple organ failure in patients. As such, blocking production of IL-18 is also a way of preventing multiple organ failure during the onset of SAP. In this study, the treatment group exhibited significantly lower levels of the inflammatory mediators mentioned before, illustrating that hydrogen-rich saline could attenuate the inflammatory response in SAP.

Hydrogen is the simplest element in nature, in addition to being reducible in diatomic gasses. In 2007, Ohsawa et al. [29] firstly reported that animals breathing 2% hydrogen could effectively eliminate oxygen radicals, as well as improve the brain's ability to concentrate and avoid damage while lacking blood flow. Buchholz et al. [30] later proved that breathing 2% hydrogen could be used to treat inflammatory response damage caused by small intestine transplants. This research illustrates that hydrogen is an ideal free radical, especially as an exterminator of poisonous free radicals. Hydrogen molecules also have excellent membrane permeability and are easily able to pass through membranes into cells and organelles. During the pathologic state, the organism will produce more oxygen radicals than it is able to dispose of, as well as cause oxidative damage to both DNA and proteins, even causing organ failure [31–33]. The results of our experiments show that severe acute pancreatitis in rats is accompanied by a decrease in amounts of SOD and GSH in the pancreatic tissue, proving that large amounts of oxygen radicals can clearly reduce SOD and GSH activity. This also means that SOD and GSH activity can reflect the antioxidant capabilities of the pancreatic ascinar cells, as when SOD and GSH levels drop, the antioxidant capabilities of the pancreas are also visibly reduced. After injection of hydrogen-rich saline, SOD and GSH levels were significantly higher than those in the treatment group, showing that hydrogen-rich saline would reduce peroxide levels, increase free-radical capabilities, and inhibit oxygen activation, thus effectively protecting pancreatic tissue. Furthermore, recent researches show that hydrogen can also have a similar effect when influencing cell signaling; thus it has been conjectured that hydrogen, after NO, CO, and H_2_S, is a new type of gas signal molecule [34].

## 5. Conclusion

This research shows that intravenous hydrogen-rich saline injections could reduce oxidative damage caused in SAP and significantly lessen the presence of inflammatory cytokines. It could also improve the antioxidative abilities of the pancreatic tissue, thus effectively protecting pancreatic tissue. Hydrogen is a kind of ideal radical scavenger, antiinflammatory, antioxidation, nonpoisonous, nonsuperfluous, conveniently priced, and with many other advantages. In the course of SAP treatment, it has wide-ranging applicability and significant future potential. At the same time, further studies are necessary to reveal the detailed mechanisms underlying the therapeutic effects of H2 and may reveal processes shared by other antioxidants that could be manipulable targets of future therapeutic strategies of SAP.

## Figures and Tables

**Table 1:** Summary of study results.

Parameters	Control group	Model group	Treatment group
TNF-*α*, pg/mL	52.1 ± 6.4	1046.4 ± 13.2*	97.1 ± 8.8^*▼*,★^
IL-6, ng/L	18.6 ± 1.43	49.3 ± 4.6*	30.7 ± 3.3^*▼*,★^
IL-18, ng/L	61.4 ± 12.7	472.4 ± 98.7*	224.1 ± 65.3^*▼*,★^
TNF-*α*mRNA	0.04 ± 0.17	1.33 ± 0.36*	0.41 ± 0.21^*▼*,★^
ICAM-1	0.93 ± 0.26	6.41 ± 0.68*	2.27 ± 0.41^*▼*,★^
MDA, nmol/mg	67.4 ± 7.1	1263.4 ± 18.2*	179.4 ± 21.8^*▼*,★^
MPO, U/g	26.1 ± 2.1	57.2 ± 4.6*	418 ± 4.9^*▼*,★^
SOD, U/mg	76.8 ± 16.6	531. ± 98.4*	197.1 ± 78.36^*▼*,★^
GSH, nmol/mg	0.07 ± 0.21	1.99 ± 0.87*	0.32 ± 0.21^*▼*,★^
MDA, nmol/mg	0.56 ± 0.33	3.9 ± 0.54*	1.86 ± 0.42^*▼*,★^
Histopathological scores	0.8 ± 0.2	12.5 ± 1.1*	7.5 ± 0.9^*▼*,★^

*P < 0.05 versus control group.

^*▼*^ *P* < 0.05 versus model group.

^★^ *P* < 0.05 versus control group.
